# Does severe acute respiratory syndrome coronavirus-2 (SARS-CoV-2) cause orchitis in patients with coronavirus disease 2019 (COVID-19)?

**DOI:** 10.1080/2090598X.2020.1798862

**Published:** 2020-08-11

**Authors:** Hassan Alkhatatbeh, Dima Alzaghari, Abdelrazaq Alkhashman, Mohammed Azab, Ghazi M. Al Edwan, Mohammad Abufaraj

**Affiliations:** a Department of Urology, Faculty of Medicine, Hashemite University, Zarqa, Jordan; b Department of Urology, Prince Hamzah Hospital, Amman, Jordan; c Faculty of Medicine, Hashemite University, Zarqa, Jordan; d Prince Hamzah Hospital, Amman, Jordan; e Department of Special Surgery, Jordan University Hospital, University of Jordan, Amman, Jordan; f Department of Urology, Medical University of Vienna, Vienna, Austria

**Keywords:** Orchitis, COVID-19, SARS-CoV-2, coronavirus

## Abstract

Objectives: To assess the prevalence of clinical orchitis in patients with coronavirus disease 2019 (COVID-19).

Patients and methods: This was a retrospective clinical observational study using data of male patients who were admitted to hospital with COVID-19 confirmed by reverse transcriptase polymerase chain reaction testing between 1 March and 4 May 2020. Patients were categorised according to age groups and disease severity. Sociodemographic information and general clinical symptoms of COVID-19 and orchitis were collected.

Results: We identified a total of 253 male patients, with a mean (range) age 43 (1–78) years. Patients were followed-up until their recovery or for 21 days. We did not observe any symptoms or signs of orchitis in any patient during follow-up across all age groups and different disease status.

Conclusion: Although we did not identify any patients with COVID-19 with symptoms or signs of orchitis, such an association cannot be excluded, and further studies are needed to validate our hypothesis and exclude any association at a molecular level.

Abbreviations: ACE2: Angiotensin-converting enzyme 2; COVID-19: coronavirus disease 2019; CRP: C-reactive protein; ESR: erythrocyte sedimentation rate; HIV: human immunodeficiency virus; IRB, Institutional Review Board; ISH, *in situ* hybridisation; RT-PCR: reverse transcriptase-PCR; SARS-CoV-2, severe acute respiratory syndrome coronavirus-2; TMPRSS2: transmembrane protease, serine 2; WBC: white blood cell

## Introduction

In December 2019, a new coronavirus named severe acute respiratory syndrome coronavirus-2 (SARS-CoV-2) was reported in Wuhan, China, which resulted in the outbreak of coronavirus disease 2019 (COVID-19). The disease then spread all over the world, with the WHO declaring COVID-19 as a pandemic on 11 March 2020 [1]. By 4 May 2020 there was >3.5 million confirmed cases and ~250 000 deaths worldwide. Jordan reported the first case of COVID-19 on 1 March 2020, and faced the crisis with a mitigation strategy, forcing a nationwide lockdown, testing and contact tracing. The Ministry of Health in Jordan dedicated special hospitals to deal with confirmed cases and admitted all patients to hospitals regardless of disease severity or the general condition of the patients. By 4 May 2020, 255 male patients had been diagnosed with COVID-19, with 2% confirmed mortality.

Angiotensin-converting enzyme 2 (ACE2) was identified as a receptor for SARS-CoV and SARS-CoV-2 [2,3]. Moreover, ACE2 is highly expressed in the testis, specifically Leydig cells, Sertoli cells, and spermatogonia. Therefore, the testis is theoretically a SARS-CoV-2 target candidate, potentially causing orchitis [4,5]. In fact, SARS-CoV had been reported to cause orchitis based on post-mortem histopathological studies. Nevertheless, no viral particles in the testis were detected in some of these studies [6–8], while others reported isolating the virus from testicular cells in post-mortem studies [9]. Moreover, some investigators hypothesised that SARS-CoV-2 could be transmitted through sexual contact based on pathogenetic similarity between SARS-CoV and SARS-CoV-2 [10].

Orchitis is an inflammatory condition that can result in testicular atrophy and subfertility [11], and one of the causes of orchitis is viral infection. Indeed, the mumps virus remains the most common cause of viral orchitis [12], and other viruses such as Epstein–Barr virus, human immunodeficiency virus (HIV), hepatitis B virus, hepatitis C virus, adenovirus and Zika virus are also reported to cause this condition. Orchitis is usually diagnosed clinically based on the presence of testicular pain and swelling. Urine culture and Doppler ultrasound are used to exclude other differential diagnoses, mainly bacterial epididymo-orchitis and testicular torsion.

In the present study, we aimed to estimate the prevalence of clinical orchitis in patients admitted with a confirmed COVID-19 diagnosis.

## Patients and methods

**Figure 1.**
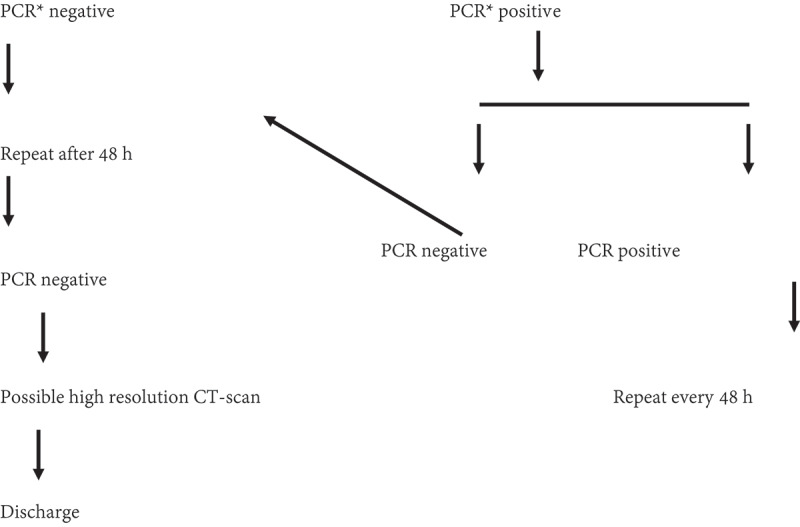
Follow-up protocol of COVID19-confirmed cases in Prince Hamzah hospital. *PCR done day 6 of admission.

This was a retrospective clinical observational study using data of 255 patients with COVID-19 from 1 March to 4 May 2020. All male patients had a confirmed COVID-19 diagnosis using real-time reverse transcriptase PCR (RT-PCR) from nasopharyngeal or oropharyngeal swabs. Two patients were excluded, as one had an active bacterial UTI and one had a previous history of recent sexually transmitted infection, leaving 253 patients for the final analysis. Complete history and physical examination were performed for all patients focussing on clinical symptoms and signs of orchitis: scrotal pain, hotness, redness or tenderness. Patients were evaluated by the urology team every 2 days until discharge from the hospital or for a total of 21 days, whichever comes first. Patients were discharged if they were confirmed to have had two negative RT-PCR results at least 48 h apart. The management protocol at our hospital is described in Figure 1. All symptomatic patients aged >18 years were given hydroxychloroquine 200 mg twice daily. Personal protective equipment with full precaution protocols were used while in contact with the patients. Urine analysis and culture were performed for all patients upon admission.

The incidence of orchitis was evaluated across different age groups and different disease severities. Patients were divided according to age into four groups: Group 1, 1–15 years (*n* = 53); Group 2, 16–40 years (*n*= 63); Group 3, 41–60 years (*n* = 77); and Group 4 > 60 years (*n*= 60). Disease severity was categorised as asymptomatic, mild or severe. Asymptomatic was defined as the absence of fever and respiratory symptoms with a normal chest radiograph. Patients with respiratory symptoms with or without fever, with or without radiographic evidence of pneumonia were considered as mild. The presence of dyspnoea or evidence of respiratory failure was defined as severe symptoms. Erythrocyte sedimentation rate (ESR), C-reactive protein (CRP), and white blood cell (WBC) count were recorded.

Descriptive statistics were used and variables were expressed as percentages, numbers or means.

This study was approved by the Institutional Review Board (IRB) at our hospital and all participants provided informed consent.

## Results

A total of 253 male patients were included in this study, with a mean (range) age of 43 (1–78) years. We evaluated the patients for a mean (range) of 15 (9–21) days. In all, 53 (21%) patients were asymptomatic and 152 (60%) had mild symptoms (Table 1). While, 48 patients (19%) had severe symptoms; of which 12 (25%) were critical and required admission to the intensive care unit. Regardless of the age of the patient or the severity of the disease, none of our patients were found to have any of the symptoms or signs of orchitis. During follow-up, 164 (65%) patients recovered and were discharged after two negative RT-PCR results and five (2%) patients died of the disease.

**Table 1.** The demographic characteristics and laboratory results in the different age groups.

	Total (*N* = 253)	Group 1 1–15 years (*n* = 53)	Group 2 16–40 years (*n* = 63)	Group 3 41–60 years (*n*= 77)	Group 4 >60 years (*n* = 60)
COVID-19					
Asymptomatic, *n* (%)	53 (21)	22 (42)	16 (26)	12 (16)	4 (7)
Mild symptoms, *n* (%)	152 (60)	31 (58)	43 (68)	47 (61)	30 (50)
Severe symptoms, *n* (%)	48 (19)	0 (0)	4 (6)	18 (23)	26 (43)
Orchitis signs or symptoms, *n*	0	0	0	0	0
CRP, mg/L, mean (range)	31.8 (0.2–264)	10.2 (0.2–28)	23.5 (1–187)	36 (2–210)	47 (4–264)
ESR, mm/h, mean (range)	25.1 (1–72)	6 (1–10)	18 (12–34)	33 (28–57)	39.6 (27–72)
WBC count, × 10^8^/L, mean (range)	11.9 (4–32)	8 (4–12)	10.8 (5.2–14)	12.5 (7–18)	15.8 (7–32)

## Discussion

Several investigators pointed out the potential effect of SARS-CoV-2 on testicular tissue based on the presence of ACE2 receptors in the testis. The authors highlighted the importance of clinical assessment of patients with COVID-19 for this possible effect [4,5]. In 2006, Xu et al. [8] published a post-mortem histopathological study in multiple organs of six patients, who died from SARS-CoV in China, and the study showed that germ cells of the testis were significantly affected with lymphocytic and macrophages infiltration and fibrosis, indicating orchitis in all specimens. Data from another post-mortem study reported focal testicular atrophy in five of seven patients without cellular infiltrate. Similar to previous reports, viral particles were not detected by *in situ* hybridisation (ISH) or electron microscopy in the testis [7]. While the authors detected the viral particles in organs such as parathyroid and pituitary glands, they did not detect it in the testis using ISH [7]. Such data might indicate an inflammatory response rather than direct viral invasion [6].

While high-dose steroids that was used during the previous epidemic might cause damage to testicular parenchyma, such therapy was not used to treat patients with COVID-19 neither worldwide nor at our institution at the time of conduction of the present study. However, Zhao et al. [9] were able to isolate the virus from testicular epithelial cells and Leydig cells in a post-mortem study in six patients who died from severe acute respiratory infection in 2003. While these studies leave theoretical concerns about the effect of COVID-19 on the testis, our present findings refuted this theory at a clinical level, as we did not observe any case of clinical orchitis in patients with COVID-19. In fact, our thorough clinical observation of 253 patients, across different age groups and severity of disease, did not show an association between COVID-19 and clinical orchitis.

To the best of our knowledge, this is the first retrospective cohort study assessing the prevalence of clinical orchitis in COVID-19-confirmed cases through frequent clinical assessment of hospitalised patients. However, there are a few recent studies investigating the presence of SARS-CoV-2 RNA in the seminal fluid of recovered COVID-19 patients [13,14]. One case report and one case series of 36 Chinese males showed that viral RNA was absent in all patients with confirmed infection. On the other hand, well-known viruses that cause orchitis such as mumps, HIV, human herpes, Ebola, and Zika were detected in the semen of infected patients [15,16].

Investigators proposed that the spike (S) protein of coronaviruses facilitates viral entry into target cells [3] depending on the binding of the S1 subunit of the S protein to the ACE2 receptor [2]. However, this attachment is not sufficient for viral entry and requires S protein priming by transmembrane protease, serine 2 (TMPRSS2), which mediates S protein cleavage at the S1/S2 site and allows fusion of viral and cellular membranes and hence viral entry into the host cell [17]. Recently, Pan et al. [13] found that ACE2 and TMPRSS2 are expressed sparsely in the human testes, with almost no overlapping gene expression. The authors concluded that ACE2-mediated viral entry of SARS-CoV-2 into target host cells is unlikely to occur within the human testicle based on an ACE2/TMPRSS2-mediated mechanism.

Our present study included a wide range of ages because all the patients with suspected COVID-19 in Jordan were hospitalised, and this allowed us to evaluate them thoroughly during their hospital stay without loss to follow-up of any of the patients and under controlled conditions. The periodic evaluation was during their hospital stay or up to a total of 21 days, as orchitis associated with other viral illnesses, such as mumps, usually occur within 1–2 weeks of the onset of symptoms.

Our present findings are significant because orchitis might result in serious complications affecting fertility. While our present data did not find an association between COVID-19 and clinical symptoms or signs of orchitis, we fill a gap in knowledge and pave the way for researchers to investigate other complications of this disease, such as the adverse impact on semen fluid analysis or fertility.

Our present study has several limitations worth mentioning. As the virus is still new and the exact pathogenesis is still under evaluation, the exact course of the orchitis may not resemble that in other viruses. In addition, we did not perform other investigations to assess the effect of the virus on reproduction, such as hormonal profile and seminal fluid analysis were not done due to the acute setting and the scope of the study to observe the clinical manifestations. Further studies might evaluate the consequences of COVID-19 on recovered patients who did not develop clinical symptoms or signs of orchitis. Moreover, the relatively small sample size might not be able to capture rare complications of COVID-19. In addition, most of our patients were asymptomatic or had mild-to-moderate symptoms (81%) and it is possible that viral threshold was not achieved to cross the blood–testis barrier, as some researchers suggested that higher viral loads are associated with more severe symptoms with more extrapulmonary involvement [18]. However, we followed-up our patients, including those with severe symptoms, thoroughly during their hospital stays and orchitis was unlikely to be missed.

## Conclusion

We did not observe symptoms or signs of orchitis in our present cohort of patients with COVID-19 across a range of age and disease severity. While our present clinical observation generates a hypothesis of a lack of association between COVID-19 and orchitis, further studies are needed to exclude the possibility of late orchitis or possible detrimental effect on spermatogenesis or hormonal function of the testis, especially in patients with more severe illness.
